# Vanillic acid attenuates Aβ_1-42_-induced oxidative stress and cognitive impairment in mice

**DOI:** 10.1038/srep40753

**Published:** 2017-01-18

**Authors:** Faiz Ul Amin, Shahid Ali Shah, Myeong Ok Kim

**Affiliations:** 1 Department of Biology, Division of Applied Life Science (BK 21), College of Natural Sciences, Gyeongsang National University, Jinju, 660-701, Republic of Korea

## Abstract

Increasing evidence demonstrates that β-amyloid (Aβ) elicits oxidative stress, which contributes to the pathogenesis and disease progression of Alzheimer’s disease (AD). The aims of the present study were to determine and explore the antioxidant nature and potential mechanism of vanillic acid (VA) in Aβ_1-42_-induced oxidative stress and neuroinflammation mediated cognitive impairment in mice. An intracerebroventricular (i.c.v.) injection of Aβ_1-42_ into the mouse brain triggered increased reactive oxygen species (ROS) levels, neuroinflammation, synaptic deficits, memory impairment, and neurodegeneration. In contrast, the i.p. (intraperitoneal) administration of VA (30 mg/kg, for 3 weeks) after Aβ_1-42_-injection enhanced glutathione levels (GSH) and abrogated ROS generation accompanied by an induction of the endogenous nuclear factor erythroid 2-related factor 2 (Nrf2) and heme oxygenase 1 (HO-1) via the activation of Akt and glycogen synthase kinase 3β (GSK-3β) in the brain mice. Additionally, VA treatment decreased Aβ_1-42_-induced neuronal apoptosis and neuroinflammation and improved synaptic and cognitive deficits. Moreover, VA was nontoxic to HT22 cells and increased cell viability after Aβ_1-42_ exposure. To our knowledge, this study is the first to reveal the neuroprotective effect of VA against Aβ_1-42_-induced neurotoxicity. Our findings demonstrate that VA could potentially serve as a novel, promising, and accessible neuroprotective agent against progressive neurodegenerative diseases such as AD.

Alzheimer’s disease (AD) is a progressive neurodegenerative disorder with no known cure that causes significant dementia in elderly people^1^. The amyloid hypothesis of AD postulates that β-amyloid (Aβ) deposition and neurotoxicity play a causative role in AD^2^. Aβ_1-42_ is neurotoxic in both *in vitro* and *in vivo* models^3^,^4^ and affects the main biochemical cascade associated with memory impairment in individuals with AD^5^. There is abundant evidence that oxidative stress is not only an early event in AD that occurs prior to changes in cytopathology but also plays a role in nerve cell dysfunction and death in AD^6^,^7^. Although the mechanisms through which Aβ exerts its toxicity are numerous and have not yet been fully elucidated, it appears that oxidative injury is an important feature in AD pathogenesis, even before the appearance of Aβ deposits^8^. Recent evidence suggests that the neurotoxic properties of Aβ are mediated by oxidative stress^9^. Oxidative stress is thought to be a key factor in the pathogenesis of AD and mild cognitive impairment^10^.

Oxidative stress is caused by an imbalance between the levels of reactive oxygen species (ROS) and a biological system’s ability to detoxify the reactive intermediates or its inability to restore the resulting damage^11^,^12^. Oxidative stress is involved in the pathophysiology of several neurodegenerative disorders characterized by progressive cognitive deficits^13^. Therefore, there is a keen interest in the introduction and development of antioxidant therapies for the treatment of cognitive decline during AD. Antioxidants are substances that can scavenge free radicals and help reduce the incidence of oxidative stress-provoked damage, as well as maintain cellular redox balance^14^,^15^. For this purpose, an increasing interest in the therapeutic use of antioxidants in the treatment of diseases associated with oxidative stress has developed^16^,^17^. The antioxidant activity of vanillic acid (VA) appears to be significant^18^. The *in vitro* antioxidant mechanisms of VA include free radical scavenging activity, reducing power, and the inhibition of lipid peroxidation^19^. Vanillic acid abolished the deleterious effect of oxidative stress induced by STZ on learning and memory in mice and exerted specific anti-inflammatory and anti-oxidant effects that down-regulated the neuroinflammatory process, suggesting that the long-term administration of VA could delay the progression of AD^20^. These findings are in line with another report^21^ describing the beneficial effects of VA. Vanillic acid has been associated with multiple pharmacologic activities such as the inhibition of snake venom activity and antifilarial, antimicrobial, anti-inflammatory and antioxidant functions^21^,^22^,^23^,^24^,^25^,^26^.

The ability of VA to ameliorate Aβ_1-42_-induced oxidative stress neuroinflammation and cognitive deficits in an AD mouse model is not known. The aims of this study were to investigate the neuroprotective efficacy of VA against Aβ_1-42_-induced oxidative stress, neuroinflammation and cognitive impairment in an *in vivo* AD mouse model.

## Results

### Beneficial effects of vanillic acid against Aβ_1-42_-induced neurotoxicity *in vitro*

**Figure 1:**
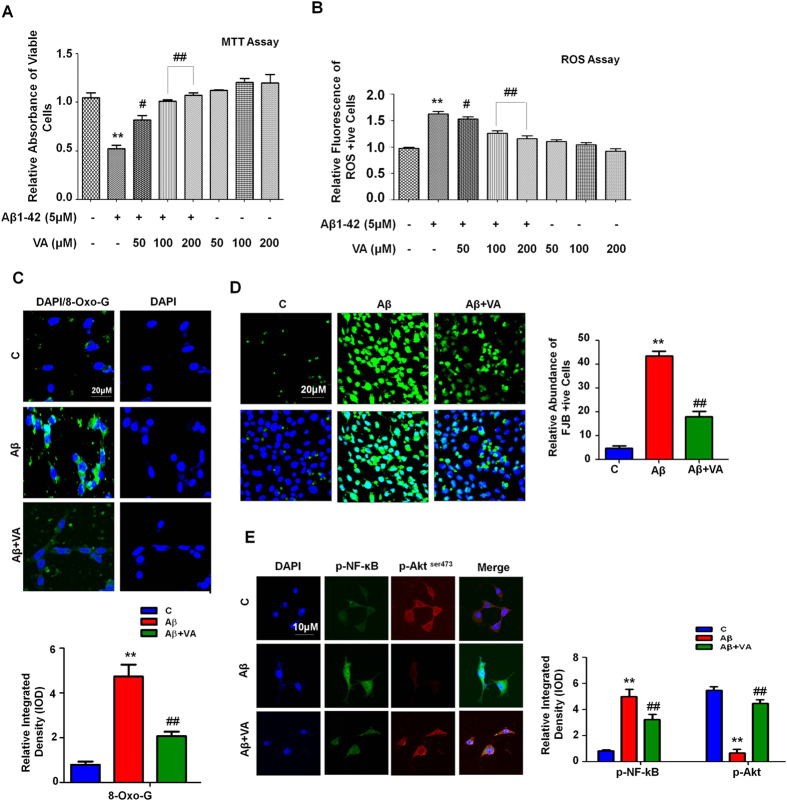
The beneficial effects of VA on Aβ_1-42_-induced neurotoxicity *in vitro*. **(A)** Shown is the cell viability (MTT assay) histogram. Aβ_1-42_ (5 μM) reduced cell viability; treatment with VA at three different concentrations (50, 100 and 200 μM) increased the viability of HT22 cells after 24 h. **(B)** Representative ROS assay histogram. Treatment with vanillic acid at all three different concentrations (50, 100 and 200 μM) significantly reduced Aβ_1-42_-induced (5 μM) ROS levels. These assays were performed in triplicate (±S.E.M.). **(C)** The immunofluorescence images along with their respective histogram of 8-OxoG (green) counterstained with DAPI (blue) in all three treated groups in HT22 cells. **(D)** Shown are the immunofluorescence images of FJB staining along with the respective integrated density histograms in HT22 cells treated with Aβ_1-42_ (5 μM) and VA (100 μM) for 24 h. **(E)** The double immunofluorescence images of HT22 cells after Aβ_1-42_ and VA treatment for 24 h, showing p-NF-kB (green), p-Akt (red), proteins and their respective relative density histograms. DAPI (blue) was used to counterstain the nucleus. These experiments were performed in triplicate. Details are given in the methods section. *Significantly different from vehicle-treated animals; ^#^significantly different from Aβ_1-42_-treated animals. Significance = **P < 0.01, ^#^P < 0.05, ^##^P < 0.01.

We determined the beneficial effects of VA against Aβ_1-42_-induced toxicity in HT22 cells. To understand the effects of VA on cell viability, HT22 cells were treated with Aβ_1-42_ (5 μM) and with three different concentrations (50, 100 and 200 μM) of VA either alone or in combination with Aβ_1-42_ for 24 h. The cell viability histogram reveals that Aβ_1-42_ induced significant (2-fold decrease) in cell viability after 24 h compared with the control cells. However, VA was non-toxic to HT22 cells at all three concentrations (50, 100 and 200 μM); similarly, VA co-treatment with Aβ_1-42_ significantly increased (1.5-, 1.9- and 2-fold respectively) cell viability (Fig. 1A).

To understand the antioxidant effects of VA against Aβ_1-42_-induced oxidative stress, we conducted fluorescence based (2′7′-dichlorodihydrofluorescein) ROS assay *in vitro*. Aβ_1-42_ (5 μM) treatment resulted in a significant increase (1.6-fold) in ROS levels compared with the control cells. In contrast, VA treatment at all three different concentrations (50, 100 and 200 μM) reduced (1.1-, 1.3- and 1.4-fold respectively) ROS levels, indicating that VA is a potent antioxidant (Fig. 1B). Additionally, VA treatment reduced (2.2-fold) the immunoreactivity of 8-Oxoguanosin (8-OxoG) against Aβ_1-42_ treated group in HT22 cells as shown in Fig. 1C.

FJB staining was performed to assess Aβ_1–42_-induced neuronal cell death morphologically. The FJB results indicate that Aβ_1-42_ (5 μM for 24 h) treatment significantly (9-fold) increased the number of dead neurons in contrast to untreated control cells (Fig. 1D). Co-treatment of VA (100 μM, 24 h) significantly reduced (2.4-fold) the number of FJB positive cells, demonstrating a protective effect of VA against Aβ_1–42_-induced neurotoxicity (Fig. 1D). Finally, the double immunofluorescence results showed that, compared with the untreated control cells, Aβ_1–42_ (5 μM) significantly increased (5.8-fold) the expression of pro-inflammatory markers such as phospho-NF-κB (p-NF-κB) and reduced (5.4-fold) phospho-Akt (Ser473) expression levels. However, VA co-treatment (100 μM) not only significantly inhibited (1.5-fold) the immunofluorescence signal of phospho-NF-κB but also increased (4.2-fold) phospho-Akt (Ser473) expression in Aβ_1–42_-treated HT22 cells (Fig. 1E).

### Vanillic acid supplementation attenuated Aβ accumulation and BACE-1 (β-site APP-cleaving enzyme-1) expression

**Figure 2:**
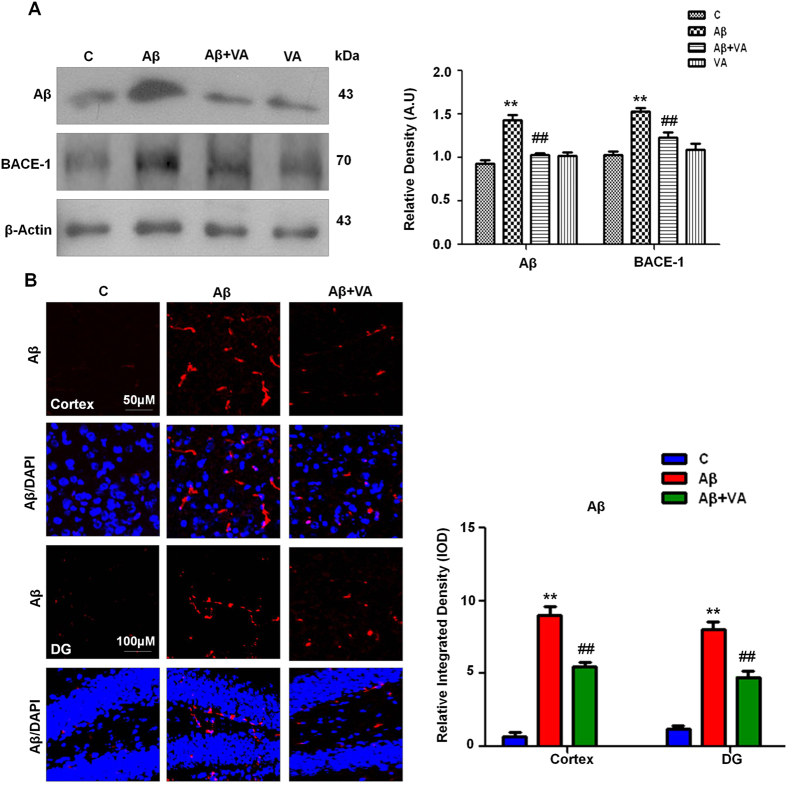
Vanillic acid attenuated Aβ accumulation and β-site APP cleaving enzyme 1 (BACE-1) overexpression in mouse brain homogenates. **(A)** Immunoblot analysis of Aβ and BACE- 1 protein expression in the mouse brain following Aβ and VA administration. The bands were quantified using Sigma Gel software, and the differences are represented by a histogram. β-Actin was used as a loading control. The density values are expressed in arbitrary units (A.U.) as the mean ± S.E.M. for the indicated protein (n ± 5 mice/group). **(B)** The immunofluorescence of Aβ was used to evaluate the cortex and hippocampus of experimental mice (n ± 8 mice/group). Magnification, 10X. *Significantly different from vehicle-treated animals; ^#^significantly different from Aβ_1-42_-treated animals. Significance  ± **P < 0.01; ^##^P < 0.01.

Antioxidants have been reported to inhibit Aβ production^27^. To determine whether the i.c.v. administration of Aβ_1-42_ promoted Aβ accumulation in the brain, we performed immunoblot and immunofluorescence analyses. Immunoblot results showed higher levels (1.5-fold) of Aβ in the Aβ_1-42_-treated mice than that in the control mice. Vanillic acid administration results in an increase accumulation of VA in the brain of mice (Suppl. Fig. 1) which attenuated the effects of Aβ_1-42_ and significantly reduced (1.4-fold) the levels of Aβ compared to the Aβ_1-42_ only mice (Fig. 2A). Similarly, BACE-1 expression was examined after Aβ_1-42_ injection, and the immunoblot results showed that Aβ_1-42_ treatment significantly increased (1.4-fold) BACE-1 expression compared to the control mice, and VA treatment significantly reduced (1.2-fold) BACE-1 expression in the Aβ_1-42_-treated mice in comparison to Aβ_1-42_-injected mice without VA treatment (Fig. 2A).

The immunofluorescence images also supported our western blot results that Aβ_1-42_-treated mice showed increased Aβ immunofluorescence reactivity in the cortex (8.5-fold) and hippocampal DG regions (6.6-fold) compared to the control group. In contrast, VA supplementation in combination with Aβ_1-42_ significantly reduced (1.6- and 1.7-fold respectively) Aβ immunofluorescence reactivity in the aforementioned regions of the mice (Fig. 2B).

### Vanillic acid abrogates Aβ_1-42_-induced oxidative stress in the mice brain via the Akt/GSK3β/Nrf2 signaling pathway

**Figure 3:**
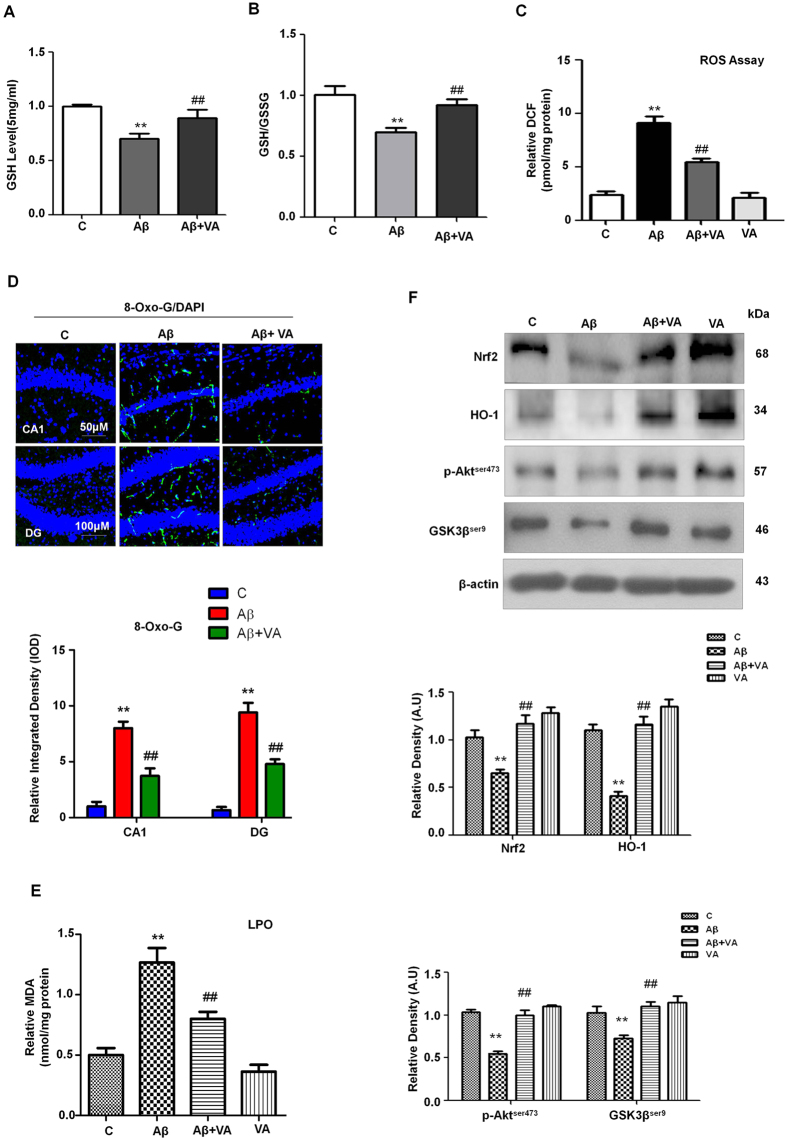
Vanillic acid treatment ameliorates ROS and oxidative stress in Aβ_1-42_-treated mice. **(A)** A representative histogram showing the ROS level in the mouse brain (n ± 5 mice/group). **(B)** A representative histogram showing GSH levels in the brains of mice. GSH levels were measured with a colorimetric assay kit and were expressed as nmol/mg protein. **(C)** A representative histogram showing the GSH/GSSG levels in the brains of mice. **(D)** The images given shows the immunoreactivity of 8-OxoG (green) along with their respective histogram counterstained with DAPI (blue) in all three treated groups in the hippocampal CA1 and DG region. **(E)** The histogram depicts the LPO levels in the treated groups. The methods details are given in the material and methods section. All these experiments were performed in triplicates. **(F)** Vanillic acid treatment stimulated the Akt/GSK3β/Nrf2/HO-1 pathway in the brains of Aβ_1-42_-treated mice. Western blot analysis demonstrated the expression of Nrf2, HO-1, p-Akt, and GSK3β in the brains of mice. The bands were quantified using Sigma Gel software, and the differences are represented in a histogram. β-Actin was used as a loading control. The density values are expressed in arbitrary units (A.U.) as the mean ± S.E.M. for the indicated proteins (n ± 5 mice/group). *Significantly different from vehicle-treated mice; ^#^significantly different from Aβ_1-42_-treated mice. Significance = **P < 0.01; ^##^P < 0.01.

Oxidative stress is implicated in various neurodegenerative diseases, including AD^28^. Our results indicated that, VA treatment markedly enhanced (1.2- and 1.3-fold respectively) GSH and GSH/GSSG levels in the brain homogenates of Aβ_1-42_-treated mice (Fig. 3A,B). To examine the beneficial effects of VA against Aβ_1-42_-induced oxidative stress in the mouse brain, an ROS assay was performed on the brain homogenates of all treated groups. The results showed that Aβ_1-42_ induced oxidative stress by significantly increasing (3.8-fold) ROS levels compared to the vehicle-treated mice, whereas VA administration in combination with Aβ_1-42_ significantly reduced (1.6-fold) ROS levels in mice (Fig. 3C). Similarly, VA treatment significantly decreased the immunoreactivity of 8Oxo-G (2-fold) and lipid peroxidation (LPO) levels (1.5-fold) in the mice hippocampus against Aβ_1-42_ injected group as evident in the Fig. 3D,E respectively.

Previous studies have demonstrated that HO-1 acts as a cellular defense mechanism in protection against ROS attack^29^. The nuclear translocation of Nrf2 and the expression of its target gene products, including HO-1, elicited an antioxidant response that may have therapeutic value for the treatment of AD^30^. Therefore, we assessed the effects of VA on the activation of Nrf2/HO-1 in the brains of Aβ_1-42_-treated mice. The western blot analyses reveal a decreased expression (1.5-fold) of Nrf2 (in the nucleus) and HO-1 (2.6-fold) proteins in Aβ_1-42_-treated mice, whereas VA treatment (30 mg/kg for 3 weeks) significantly increased (1.7-fold) the expression of Nrf2 (in the nucleus) and HO-1 (2.8-fold) in the brains of Aβ_1-42_-treated mice. (Fig. 3F).

Recent studies have suggested that the Akt/GSK-3β signaling pathway is involved in the nuclear translocation of Nrf2^31^,^32^. The phosphorylation of GSK-3β (Ser9) leads to the inactivation of GSK-3β activity. In the current study, we found that Akt phosphorylated at serine 473 (p-Akt ser473) and GSK-3β phosphorylated at serine 9 (p-GSK-3β ser9) were significantly reduced (1.9- and 1.4-fold) in Aβ_1-42_ treated mice compared with control mice. Conversely, VA treatment significantly increased (1.8-fold) p-Akt and p-GSK-3β (1.7-fold) expression levels in the Aβ_1-42_ treated mice (Fig. 3F).

### Vanillic acid treatment attenuated Aβ_1-42_-induced glial cell activation (microglia and astrocytes) and neuroinflammation in the mouse brain

**Figure 4:**
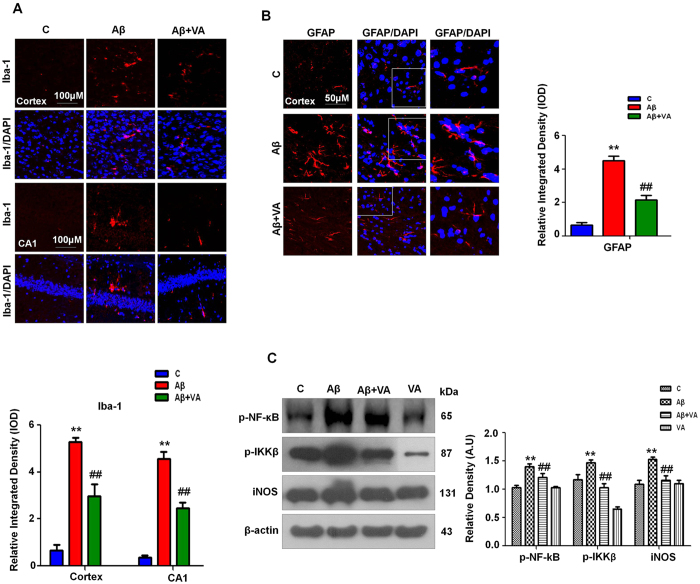
Vanillic acid treatment attenuated the number of Aβ_1-42_-induced activated glial cells (microglia and astrocytes) and reduced neuroinflammation in the mouse brain (n ± 8 mice/group). **(A)** Iba-1 immunofluorescence revealed a significant increase in the number of Iba-1 reactive cells in the brains of mice in the Aβ_1-42_-treated group compared to mice in the vehicle-treated group. On the other hand, VA treatment significantly decreased the number of reactive Iba-1 cells in the brains of mice exposed to Aβ_1-42_. **(B)** Immunofluorescence images revealed a significant increase in the number of GFAP reactive cells in the brains of mice in the Aβ_1-42_-treated group compared to the vehicle-treated group. Vanillic acid treatment significantly decreased the number of reactive GFAP cells in the brains of mice exposed to Aβ_1-42_. **(C)** Western blot analysis of p-IKKβ, p-NF-κB and iNOS in the brain of mice. The bands were quantified using Sigma Gel software, and the differences are represented in a histogram. β-Actin was used as a loading control. The density values are expressed in arbitrary units (A.U.) as the mean ± SEM for the indicated proteins (n ± 5 mice/group). *Significantly different from vehicle-treated mice; ^#^significantly different from Aβ_1-42_-treated mice. Significance = **P < 0.01, ^##^P < 0.01.

Several studies have indicated that there is an increase in microgliosis and astrocytosis in old age that can contribute to neurological disorders such as AD^33^,^34^. Glial fibrillary acidic protein (GFAP) and ionized calcium-binding adaptor molecule 1 (Iba-1) are specific markers for activated astrocytes and microglia, respectively. Therefore, we investigated the protective effect of VA against microglial (Iba-1 reactive cells) and astrocyte (GFAP reactive cells) activation. Immunofluorescence images in cortex and hippocampus revealed a significant increase in the number of Iba-1 and GFAP (7.9- and 6.8-fold respectively) reactive cells in the brains of mice in the Aβ_1-42_-treated group compared to vehicle-treated mice. On the other hand, VA treatment significantly decreased the number of reactive Iba-1 (1.8-fold) and GFAP (2.1-fold) cells in the brain in the Aβ_1-42_-treated group (Fig. 4A,B).

NF-κB expression has been reported to increase during aging^35^. Similarly Terai *et al*. reported that NF-κB has been found in neurons and neurofibrillary tangles in the brain of patient with AD after postmortem^36^. Western blot results revealed that the expression of p-NF-κB and phospho-IKK-β was increased (1.3- and 1.2-fold respectively) in the brains of Aβ_1-42_-treated mice compared to mice in the vehicle-treated group. Vanillic acid treatment (30 mg/kg for 3 weeks) significantly reduced the expression of p-NF-κB (1.1-fold) and p-IKK-β (1.4-fold) in Aβ_1-42_-treated mice (Fig. 4C). Similarly, NF-κB activation could lead to the activation of various pro-inflammatory markers that are implicated in neuronal degeneration^37^. The levels of activated inflammatory markers such as inducible nitric oxide synthase (iNOS) were analyzed in Aβ_1-42_-treated brains via western blot analysis. The results revealed that VA significantly reduced (1.3-fold) Aβ_1-42_-induced neuroinflammation by inhibiting iNOS expression in the mouse brain (Fig. 4C).

### Vanillic acid rescued the mouse brain against Aβ_1-42_-induced neuronal apoptosis and neurodegeneration

**Figure 5:**
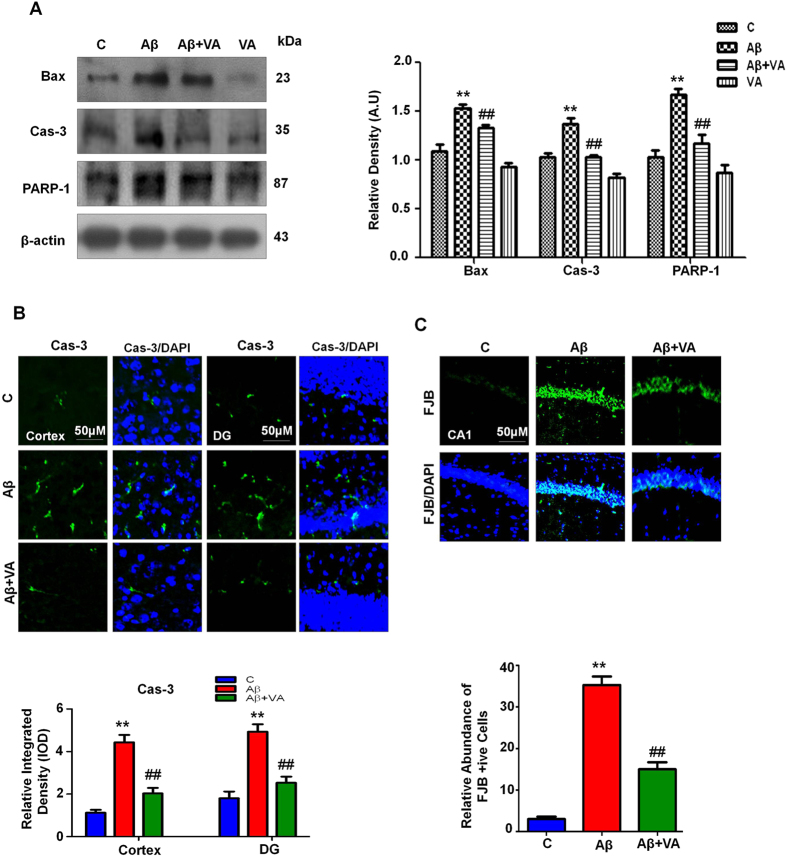
Vanillic acid prevented Aβ_1-42_-induced apoptosis and neurodegeneration. **(A)** Immunoblot analysis of the mouse brain using activated Bax, caspase-3 and cleaved PARP-1 antibodies. The bands were quantified using Sigma Gel software, and the differences are represented in a histogram. Anti-β-actin was used as a loading control. The density values are expressed in arbitrary units (A.U.) as the mean ± S.E.M. for the indicated brain proteins (n ± 5 mice/group). **(B)** Immunofluorescence of activated caspase-3 in the cortex and hippocampus in the experimental mice (n ± 8 mice/group). Caspase-3-positive neurons were increased in the Aβ_1-42_-treated mice compared with the control mice. Vanillic acid treatment significantly decreased the number of Aβ_1-42_-induced caspase-3-positive neurons. **(C)** Immunofluorescence of FJB positive neurons in the CA1 region of vehicle, Aβ_1-42_ and VA treated mouse brains. Magnification, 10X. *Significantly different from the vehicle-treated; ^#^significantly different from Aβ_1-42_ treated. Significance = **P < 0.01 and ^##^P < 0.01.

Pro-apoptotic Bax and anti-apoptotic Bcl-2 are members of the Bcl-2 family and are the main regulators of the apoptotic pathway in mitochondria^38^. Our immunoblot results indicate that Aβ_1-42_ caused the upregulation of Bax proteins (1.3-fold) and VA treatment significantly reduced (1.1-fold) the expression of Bax in mice treated with Aβ_1-42_ (Fig. 5A).

Previous reports have determined that Aβ_1-42_ plays a critical role in inducing apoptotic neurodegeneration in AD^39^. Aβ-induced neuronal apoptosis is mediated via the apoptotic caspase cascade, which includes caspase-9 and caspase-3^40^. We investigated the levels of caspase-3 in response to Aβ_1-42_ and VA treatment via western blot and immunofluorescence analysis. Our results (both western blot and immunofluorescence, Fig. 5A,B) showed higher (1.3- and 3.9-fold respectively) levels of activated caspase-3 in Aβ_1-42_-treated mice compared to the control group. Treatment with VA attenuated the Aβ_1-42_-induced expression of active caspase-3 and significantly decreased (1.3- and 2.2-fold respectively) the level of caspase-3 compared to the mice treated with Aβ_1-42_ alone (Fig. 5A,B).

Poly (ADP-ribose) polymerase-1 (PARP-1) is involved in DNA repair, and the overexpression of PARP-1 due to the exposure to different excitotoxic agents induces neurodegeneration^41^. Aβ_1-42_-peptide has been demonstrated to trigger the overactivation of PARP-1 in the adult rat hippocampus^42^. Western blot analysis revealed that PARP-1 cleavage increased (1.5-fold) in the Aβ_1-42_-treated mice, whereas the expression level of cleaved PARP-1 was significantly reduced (1.3-fold) in VA treated mice (Fig. 5A). Furthermore, the FJB results indicate that Aβ_1-42_ treatment significantly increased (3.8-fold) the number of dead neurons in contrast to untreated mice group (Fig. 5C). While treatment of VA significantly reduced (2.3-fold) the number of FJB positive neurons in mice hippocampus, demonstrating a protective effect of VA against Aβ_1–42_-induced toxicity (Fig. 5C).

### Vanillic acid treatment alleviated Aβ_1-42_-induced synaptotoxicity

**Figure 6:**
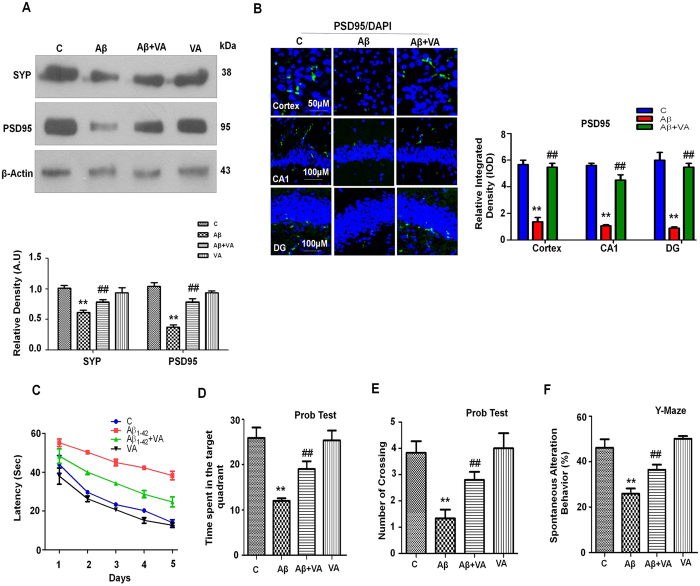
Vanillic acid treatment attenuated Aβ_1-42_-induced synaptic disorganization and cognitive impairment in mice. **(A)** Immunoblot analysis of synaptophysin and PSD95 in the mouse brain. The bands were quantified using Sigma Gel software, and the differences are represented in a histogram. β-Actin was used as a loading control. The density values are expressed in arbitrary units (A.U.) as the mean ± S.E.M. for the indicated proteins (n ± 5 mice/group). **(B)** A representative immunofluorescence image of PSD95 reactivity in the cortex and hippocampus of experimental mice (n ± 8 mice/group). Magnification, 10X. **(C)** A representative histogram for the mean escape latency (sec) in the MWM test during the training session (n ± 13 mice/group). **(D)** Time spent in the target quadrant during the probe test. **(E)** The number of platform crossings during the probe test. **(F)** A histogram showing the percentage spontaneous alterations in the Y-maze test (n = 13 mice/group). *Significantly different from the vehicle-treated mice; ^#^significantly different from Aβ_1-42_ treated mice. Significance = **P < 0.01, ^##^P < 0.01.

To analyze the protective effect of VA against Aβ_1-42_, we assessed pre- and post-synaptic protein markers. Immunoblot results showed a lower level (1.6- and 2.8-fold respectively) of the presynaptic vesicle membrane protein synaptophysin (SYP) and the post-synapse density (PSD95) protein in Aβ_1-42_-treated mice compared to the control group. Vanillic acid administration reversed the synaptotoxic effects of Aβ_1-42_ and significantly increased the expression of the SYP (1.2-fold) and PSD95 (2.1-fold) proteins in comparison to Aβ_1-42_ treated mice (Fig. 6A). Additionally, immunofluorescence results showed that Aβ_1-42_-treatment decreased (4.3-fold) the immunofluorescence reactivity of PSD95 in cortex and hippocampus compared to the control mice. Vanillic acid treatment reversed the effects of Aβ_1-42_ and significantly increased (4.1-fold) the immunofluorescence reactivity of PSD95 in the cortex and the hippocampal CA1 and DG regions (Fig. 6B).

### Vanillic acid treatment ameliorates Aβ_1-42_-induced memory impairment

Performance in the Morris water maze has been shown to be a reliable and noninvasive test to determine cognitive changes in the AD mouse model^43^. To assess whether VA could counteract Aβ_1-42_-induced memory impairment, we administered the MWM and Y-maze tests. We first recorded the learning ability of the mice (n = 13 mice/group) in the MWM test. We observed that Aβ_1-42_-treated mice showed an increased latency to reach the platform, and the mice that had received VA treatment (30 mg/kg, i.p., 3 weeks) showed a decreased escape latency (Fig. 6C). Twenty-four hours after the 5 day training session, we removed the platform and allowed the mice to swim freely. We observed that Aβ_1-42_-treated mice spent less time in the target quadrant and exhibited fewer platform crossings, revealing that Aβ_1-42_ caused memory impairment. Vanillic acid treatment improved Aβ_1-42_-induced memory impairment by significantly increasing (1.5- and 2.1-fold respectively) the time spent in the target quadrant and the number of platform crossings (Fig. 6D,E).

Following the MWM analysis, we evaluated the spontaneous alteration behavior percentage (%) of mice (n  ±  13 mice/group), observing the average total number of arm entries and successive triplets using a Y-maze test. Spontaneous alteration behavior, indicating spatial working memory, is a form of short-term memory. After the single injection of Aβ_1-42_, the % of spontaneous alternation behavior was lower (1.8-fold) in Aβ_1-42_-treated mice compared to the control mice, suggesting that Aβ_1-42_ was responsible for the decline in cognition. Treatment with VA significantly increased (1.4-fold) the spontaneous alternation behavior % in Aβ_1-42_-treated mice compared to mice that had received Aβ_1-42_ alone (Fig. 6F), indicating that VA treatment ameliorated Aβ_1-42_-induced memory dysfunction in Aβ_1-42_-treated mice.

## Discussion

Oxidative stress has been shown to contribute to AD neuropathology^44^. Increased levels of oxidative stress markers were found in neurons surrounding amyloid deposits in transgenic mouse models of the disease^45^, and the experimental induction of oxidative stress leads to Aβ accumulation in primary neurons^46^. ROS can oxidize proteins, lipids, and DNA, and increased levels of specific oxidative markers and redox metals were found in the brains of AD patients^47^. Senile plaque formation in specific regions of the brain induces neuroinflammation and free radical induction that contribute to the destruction of brain areas such as the amygdala, hippocampus, and cortex^48^. Reducing oxidative damage in the brain can be considered a promising strategy for therapeutic intervention in AD^49^. The antioxidative activity of vanillic acid is well known from *in vitro* experiments^50^,^51^.

The present study is the first to provide evidence that VA administration (30 mg/kg, i.p., 3 weeks) attenuates Aβ_1-42_-induced ROS, memory impairment, synaptic deficits, neuroinflammation and neurodegeneration in a mouse Aβ_1-42_ model. So far, the possible effects of VA have not been studied in an AD model that exhibits amyloidosis and oxidative stress. In the present study, we found that VA significantly ameliorates cognitive deficits accompanied by increased levels of GSH in brain tissues and increased Nrf2/HO-1 expression in Aβ_1-42_-treated mice. Vanillic acid exerts beneficial therapeutic effects via positively regulating Akt/GSK-3β/Nrf2 signaling pathways. Furthermore, we also determined that VA is beneficial against Aβ_1-42_-induced neurotoxicity in the neuronal HT22 cell line *in vitro*.

The administration of Aβ in mice triggered oxidative stress by increasing ROS level, memory dysfunction, synaptic disorganization (a key feature of early phase AD), neuroinflammation and potentially neuronal degeneration. In the advancement and evaluation of therapeutic strategies for AD pathology, the i.c.v. Aβ_1-42_-infusion model is a useful complement to transgenic mouse models^52^, although the mechanisms that underlie many features of AD, including synaptotoxicity, the hyperphosphorylation of tau, apoptosis, and neurodegeneration are still not clearly known. In AD, Aβ production and aggregation in the human brain has been associated with neuronal dysfunction and memory disorders^53^. BACE-1 is the primary initiating enzyme, and its activity is the rate-limiting step in APP processing and Aβ production^54^. The elevated expression of activated BACE-1 has been examined in the brain during late-onset sporadic AD, which is associated with neuronal loss and spatial memory impairment in 5XFAD APP/PS1 mice^55^. Aβ levels and Aβ_1-42_-induced BACE-1 expression in the Aβ_1-42_-treated mice were alleviated with VA treatment (Fig. 2A,B).

Previous studies involving *in vivo* and *in vitro* experiments showed that Aβ increases oxidative damage^56^. Our data in the present study showed that Aβ_1-42_-treated mice exhibited a significant increase in oxidative stress compared to WT mice. Our observations are consistent with other prior reports^57^. Vanillic acid exhibited protective effects due to its free radical scavenging, antioxidant and anti-inflammatory effects^58^. Interestingly, in this work, we found that the enhancement of oxidative stress in the brain of Aβ_1-42_-treated mice was significantly attenuated by VA treatment, which reduced ROS induction and prevented the depletion of endogenous reduced glutathione (GSH) levels, suggesting that antioxidant activity might play some role in the beneficial effects of vanillic acid in Aβ_1-42_-treated mice. Increased levels of reduced glutathione revealed that there was less radical formation, and consequently less oxidized glutathione was formed. This finding clearly revealed the antioxidant effects of VA.

A growing body of literature suggests that the activation of Nrf2 provides neuroprotection in AD^59^. The Nrf2 antioxidant pathway was impaired in transgenic AD mice concomitantly with an increased brain Aβ burden^60^. Previous work by Choudhry showed a 50% reduction in Nrf2 levels in transgenic AD mice^61^. The induction of the Nrf2 pathway by small-molecule compounds protects against neuronal oxidative stress and toxicity induced by Aβ *in vitro*^62^. A decrease in Nrf2 protein expression was observed in the brains of Aβ_1-42_-treated mice, and VA treatment significantly increased Nrf2 protein expression. Although the nuclear translocation of Nrf2 is responsible for the induction of HO-1 expression, it is uncertain whether the VA-induced enhancement in HO-1 expression contributes to the improved cognitive functions in Aβ_1-42_ mice. HO-1 is thought to be highly associated with AD pathology and is expressed in the hippocampus of patients with AD^63^. The upregulation of HO-1 has therapeutic potential for antioxidant function in AD^64^. Although HO-1 expression is known to correlate with oxidative stress, it is uncertain whether increased HO-1 levels are associated with the improvement of cognitive functioning resulting from antioxidant treatment. We observed that Aβ_1-42_-treated mice exhibited decreased HO-1 expression. Treatment with VA increased the expression of HO-1 in the brains of Aβ_1-42_-treated mice.

GSK-3β negatively regulates Nrf2 by controlling its subcellular distribution^65^. Prolonged oxidative stress, as in cases of AD, causes the inactivation of Akt, the activation of GSK-3β and the translocation of Nrf2 from the nucleus to the cytosol, thus limiting the antioxidant response of cells^31^,^32^. In agreement with previous studies on AD patients and AD mouse models, the current study shows that GSK-3β expression was decreased in the brains of Aβ_1-42_-treated mice, and VA treatment increased the expression of Akt and GSK-3β. Although the causal relationship remains unclear, it is conceivable that improved spatial learning and memory could be attributed to the VA -induced activation of the Akt pathway.

Maqbool *et al*. demonstrated that activated IKK-β/NF-κB-induced neuroinflammation promotes neurodegeneration during aging. In this study, we investigated whether Aβ_1-42_ increased the expression of IKK-β/NF-κB, which can trigger other inflammatory mediators^66^. Vanillic acid treatment reversed the Aβ_1-42_-induced elevated expression of IKK-β/NF-κB and reduced the activity of IKKβ/NF-κB. Studies have reported that activated NF-κB increased the expression of other inflammatory mediators such as iNOS2, which might be involved in the induction of memory impairment. Previous studies have described the anti-inflammatory properties of phenolic acids, VA and protocatechuic acids^67^.

We also found that the Aβ_1-42_-induced expression of these inflammatory markers might occur through the activation of the IKK-β/NF-κB pathway or astrocytic and microglial activation. Treatment with VA decreased the expression of these inflammatory markers, preventing neuroinflammation and memory impairment in the Aβ_1-42_-treated mice.

Numerous mechanisms have been associated with Aβ induced apoptosis and neurodegeneration in both *in vivo* and *in vitro* models of AD^39^,^68^. Our results also revealed that Aβ_1-42_ activates caspases. Activated caspase-3 cleaves PARP-1, leading to apoptosis and neurodegeneration^69^. The overactivation of PARP-1 is involved in NAD+ depletion, leading to neuronal cell death^70^. Vanillic acid exerted protective effects on lipids, Bax, Bcl-2 and myocardial infarct size in isoproterenol-induced rats^58^. Our results also showed that VA decreased the expression of activated caspases, which prevented PARP-1 cleavage and reduced the expression of Bax, indicating that VA prevents Aβ_1-42_-induced apoptotic neuronal cell death in Aβ_1-42_-treated mice.

Landmark studies have described Aβ-induced synaptic loss and disorganization in an animal model of AD^71^. However, the underlying mechanism of synaptic loss and disorders is still unknown. The expression of the presynaptic marker synaptophysin was decreased in the brains of patients with AD and in an Aβ animal model of AD^72^. Our results revealed that Aβ_1-42_-treated mice exhibited significantly lower synaptophysin levels in the brain. Moreover, the decreased level of the postsynaptic protein marker PSD95 was also observed in the Aβ model of AD^71^. It has been reported that PSD95 and SNAP-23 regulate AMPARs^73^. Thus, decreased levels of synaptophysin and PSD95 are associated with memory dysfunction; in Aβ_1-42_-treated mice, the levels of synaptophysin and PSD95 were enhanced after VA administration, suggesting that the prevention of synaptic disorganization through various pre- and postsynaptic (LTP)-related protein markers improve memory function.

In the current study, our results showed a significant reduction in memory function as evidenced by MWM and Y-Maze test performance. We also observed that VA treatment (30 mg/kg, i.p., 3 weeks) improved memory, as shown by the reduction in escape latency, the increased amount of time spent in the target quadrant and the number of platform crossings during the probe test. In the Y-maze, we observed a lower percentage of spontaneous alternation behavior, which is related to the function of the hippocampus^74^. Vanillic acid treatment ameliorated the effects of Aβ_1-42_ on spontaneous alternation behavior and reduced the degree of spatial memory impairment. The observed improvement in memory function associated with VA treatment demonstrates its neuroprotective effect against Aβ_1-42_-induced memory impairment.

## Conclusion

In summary, our results demonstrated that the reversal of cognitive deficits by VA treatment in Aβ_1-42_-treated mice might result from the antioxidant activity of VA; vanillic acid treatment was associated with an increased expression of HO-1, which is mediated by the activation of Akt/GSK-3β/Nrf2 signaling. This unique mechanism explains, at least partially, the potent antioxidant capacity of VA, which might allow VA to succeed in treating AD where other ‘regular’ antioxidants have failed. In addition, our results do not exclude the possible involvement of any other mechanisms in the inhibition of oxidative stress by VA. Therefore, VA could be a potential candidate for further preclinical studies aimed at the treatment of cognitive impairment and dementia.

## Materials and Methods

### Chemicals

Vanillic acid, Aβ_1-42_ peptides, DCFDA and were purchased from Sigma Aldrich (St. Louis, MO, USA). MTT and dimethyl sulfoxide (DMSO) were purchased from Promega (Madison, WI, USA).

### Cell viability assay

The viability of HT22 cells (purchased from Korean Cell Bank, Korea) was assessed with the MTT assay according to the manufacturer’s instructions (Sigma Aldrich). Briefly, the cells were cultured in 96-well plates at a density of 1 × 10^4^ cells per well in 100 μL of Dulbecco’s modified Eagle’s medium (DMEM from Gibco Life Technologies, USA). After 24 h, the medium was replaced with fresh medium containing Aβ_1-42_ (5 μM), with three different concentrations (50, 100 and 200 μM) of VA either alone or in combination with Aβ_1-42_ (5 μM), and the cells were incubated for an additional 24 h. The control cells received only DMEM. Following this, the cells were incubated with MTT solution for another 2–4 h at 37 °C. Subsequently, the medium in each well was replaced with DMSO. Finally, the absorbance of the solution in each well at 570 nm was measured using an ApoTox (Promega) instrument. All experiments were performed independently in triplicate.

### Oxidative stress (ROS) detection *in vitro*

The ROS assay was conducted in HT22 cells as described previously^75^. The cells were cultured in 96-well plates. After 24 h of incubation at 37 °C in a humidified atmosphere of 5% CO_2_, the cells were treated with fresh medium containing Aβ_1-42_ (5 μM), with or without VA at three different concentrations (50, 100 and 200 μM), and the cells were incubated for an additional 24 h. The control cells received only DMEM. Following this, a 600-μM solution of DCFDA (20, 70-dichlorofluorescein diacetate) dissolved in DMSO/PBS was then added to each well, and the cells were incubated for 30 min. The plates were then read on an ApoTox-Glo (Promega) instrument at 488/530 nm.

### Cell treatments for the immunofluorescence assay

The HT22 cells were cultured for 24 h in four-well chamber slides in DMEM and then for 12 h in fresh DMEM containing Aβ_1-42_ (5 μM) or Aβ_1-42_ (5 μM) plus VA (100 μM). Following this, the cells were washed with 0.01 M phosphate buffered saline (aMResco, Life Sciences, USA) and fixed with ice-cold 4% paraformaldehyde (NBP) for 30 min. The slides containing the fixed HT22 cells were used for the immunofluorescence assay described below.

### Animals

Male wild-type C57BL/6 N mice (25–30 g, 8 wks old, n ± 13 mice/group) were purchased from Samtako Bio (Osan, South Korea). The mice were acclimatized for 1 week in the university animal house under a 12-h/12-h light/dark cycle at 23 °C with 60 ± 10% humidity and were provided with food and water ad libitum. All of the methods and experimental procedures were conducted according to the approved (Approval ID: 125) guidelines and regulations by the animal ethics committee (IACUC) of the Division of Applied Life Sciences, Department of Biology at Gyeongsang National University, South Korea.

### Drug treatment protocol

Human Aβ_1-42_ peptide was prepared as a stock solution at a concentration of 1 mg/mL in sterile saline solution, followed by aggregation via incubation at 37 °C for 4 days. The aggregated Aβ_1-42_ peptide or vehicle (0.9% NaCl, 3 μL/ 5 min/mouse) was stereotaxically administered into the ventricle (i.c.v.) using a Hamilton microsyringe (−0.2 mm anterioposterior (AP), 1 mm mediolateral (ML), and −2.4 mm dorsoventral (DV) to Bregma) under anesthesia in combination with 0.05 mL/100 g body weight Rompun (Xylazine) and 0.1 mL/100 g body weight Zolitil (Ketamine). The rest of the protocol was the same as previously reported^76^.

Twenty-four hours after Aβ_1-42_ and vehicle i.c.v. injection, the mice were divided into the following groups: (1) control (C) mice injected i.c.v. with 0.9% saline as a vehicle or i.c.v. with Aβ_1-42_ (Aβ_1-42_ group), (2) mice injected with Aβ_1-42_ and VA 30 mg/kg intraperitoneally (i.p.) for 3 wks (Aβ_1-42_+ VA), and (3) mice treated with VA 30 mg/kg (i.p.) for 3 wks alone (VA). Twenty-four hours post i.c.v. Aβ_1-42_ or vehicle injection, VA (30 mg/kg) was administered (i.p.) daily for 3 weeks.

### Morris water maze (MWM) test

Behavior was assessed using a MWM and a Y-maze test (n ± 13 mice/group). The experimental apparatus consisted of a circular water tank (100 cm in diameter, 40 cm in height), containing water (23 ± 1 °C) at a depth of 15.5 cm, which was made opaque by adding white ink. A transparent escape platform (10 cm in diameter, 20 cm in height) was hidden 1 cm below the water surface and was placed at the midpoint of one quadrant. Each mouse received training for five consecutive days using a single hidden platform in one quadrant with three quadrants of rotational starting. Latency to escape from the water maze (finding the hidden escape platform) was calculated for each trial. Twenty-four hours after the 5th day, the probe test was performed for the evaluation of memory consolidation. The probe test was carried out by removing the platform and allowing each mouse to swim freely for 60 s. The time that mice spent in the target quadrant and the number of times the mouse crossed over the platform location (where the platform was located during hidden platform training) was measured. Time spent in the target quadrant was considered to represent the degree of memory consolidation. All data were recorded using video-tracking software (SMART, Panlab Harvard Apparatus; Bioscience Company, Holliston, MA, USA).

### Y-maze test

The Y-maze was made from black-painted wood. Each arm of the maze was 50 cm long, 20 cm high and 10 cm wide at the bottom, and 10 cm wide at the top. Each mouse was placed at the center of the apparatus and was allowed to move freely through the maze for three 8-min sessions. The series of arm entries was visually observed. Spontaneous alteration was defined as the successive entry of the mice into the three arms in overlapping triplet sets. The alteration behavior percentage (%) was calculated as [successive triplet sets (entries into three different arms consecutively)/total number of arm entries-2] × 100. A higher percentage of spontaneous alternation behavior was considered to indicate enhanced cognitive performance.

### Protein extraction from mouse brains

The mice were killed and the brains (hippocampus) were immediately removed, and the tissue was frozen on dry ice and stored at −80 °C. The brains tissues were homogenized in pro-prep^TM^ protein extraction solution according to the provider instructions (iNtRON Biotechnology, Inc., Sungnam, South Korea). The samples were then centrifuged at 13,000 r.p.m. at 4 °C for 25 min. The supernatants were collected and stored at −80 °C.

### Western blot analysis

Western blot analysis was performed according to previously reported methods with minor modifications^77^. Briefly, the animals (n ± 5 mice/group) were sacrificed and the protein concentration was measured (Bio-Rad protein assay kit, Bio-Rad Laboratories, CA, USA). Equal amounts of proteins (25–30 μg) were subjected to electrophoresis on 4–12% Bolt™ Mini Gels (Novex; Life Technologies, Kiryat Shmona, Israel). The membranes were blocked in 5% (w/v) skim milk to reduce the nonspecific binding and were then incubated with the primary antibodies (1:1000 dilution) overnight at 4 °C. After incubating with a horseradish peroxidase-conjugated secondary antibody, the specific immunocomplexes were detected using an ECL detection reagent according to the manufacturer’s instructions (Amersham Pharmacia Biotech, Uppsala, Sweden). The X-ray films were scanned, and the optical densities of the bands were analyzed by densitometry using the computer-based Sigma Gel program, version 1.0 (SPSS Inc., Chicago, IL, USA).

### Antibodies

The following antibodies were used in this study: anti-p-Akt, anti-p-NF-κB 65, anti-iNOS, anti-caspase-3, anti-PARP-1, anti-Bax, anti-Aβ, anti-BACE-1, anti-GFAP, anti-Iba-1, anti-pGSK3^ser9^, and anti-β-actin (Santa Cruz Biotechnology), anti-Synaptophysin and anti-PSD95 (Cell Signaling).

### ROS assay *in vivo*

ROS activity was assessed as described previously with some modification^78^. The assay was based on the oxidation of 2′7′-dichlorodihydrofluorescein diacetate (DCFH-DA) to 2′7′ dichlorofluorescein (DCF). Brain (hippocampus) homogenates were diluted with ice-cold Lock’s buffer at 1:20 to yield the final concentration of 2.5 mg tissue/500 μL. The reaction mixture of Lock’s buffer (1 mL, pH ± 7.4), 0.2 mL of homogenate, and 10 mL of DCFH-DA (5 mM) was incubated at room temperature for 15 min to convert DCFHDA to the fluorescent product DCF. The conversion of DCFH-DA to the DCF was assessed using a spectrofluorimeter with excitation at 484 nm and emission at 530 nm. For background fluorescence (conversion of DCFH-DA in the absence of homogenate), we measured parallel blanks. ROS quantification was expressed as pmol DCF formed/mg protein.

### GSH and GSH/GSSG assay

The levels of GSH and GSH/GSSG in the brain tissue homogenates of hippocampus region were determined by using the commercially available Glutathione Assay Kit (BioVision’s Catalog #K264-100) according to the provided protocol. This BioVision’s Glutathione Detection Kit provides a unique, convenient tool for detecting GSH, GSSG, and total glutathione separately. Briefly, in the assay, OPA (o-phthalaldehyde), reacts with GSH (not GSSG), generating fluorescence, so GSH can be specifically quantified. Adding a reducing agent converts GSSG to GSH, so (GSH + GSSG) can be determined. To measure GSSG specifically, a GSH Quencher is added to remove GSH, preventing reaction with OPA (while GSSG is unaffected). Reducing agent is then added to destroy excess quencher and convert GSSG to GSH. Thus, GSSG can be specifically quantified.

### Determination of Lipid Peroxidation

Quantification of lipid peroxidation (LPO) is essential to assess the oxidative stress. Free malondialdehyde (MDA), a marker of LPO, was measured in the tissue homogenate of the hippocampus region using lipid peroxidation (MDA) colorimetric/fluorometric assay kit (BioVision, USA, Cat# K739-100) according to the manufacturer’s protocol.

### HPLC Analysis of vanillic acid Levels in the Brain

Quantitative analysis of vanillic acid was performed by using HPLC (Perkin–Elmer 200 series, Perkin–Elmer Co., Bridgeport, USA). Separation was achieved using Zorbax bonus RP C-18 column (4.6 × 150 mm) 5 lM, Agilent, USA, at room temperature of 25 °C. The mobile phase consisted of (A) water having 0.1% analytical grade acetic acid, and (B) 100% acetonitrile. Elution was carried out in a binary gradient mode in ratio of [A:B 80:20 (5 min), 50:50 (5 mint), 30:70 (6 mint)] having flow rate 1 ml/min. The whole procedure was run for 16 mints, while chromatograms were acquired at 260 nm in UV detector. Stock solution of vanillic acid was prepared in acetonitrile at 1.0 mg/mL and stored at −20 °C. From this stock solution, 50 μg/mL working stocks and subsequent working solutions of appropriate concentrations were prepared in ACN. From the working solutions, calibration samples were prepared. After that Calibration curve was constructed using the following concentrations: 6.25, 12.5, 25, and 50 μg/mL and their respective detected peak area of each concentrations, which were fitted using least squares linear regression correlation analysis. The areas of individual peaks were calculated from extracted LC chromatograms of the given compound. The plotted four point’s calibration curves were linear having correlation coefficients higher than 0.999 for the said compound, indicating a good linearity in the proposed investigation range.

In order to determine the levels of vanillic acid in the brain, at 1 h after the last i.p. injection, the brains were rapidly removed, weighed, and washed in ice cold 0.9% NaCl. The brain was minced with scissors and placed in a homogenizer vessel; 5.5 mL acetonitrile was added and tissue was subsequently homogenized. The homogenized samples were transferred to 50 mL conical glass tubes and vortexed for 5 min prior to centrifugation at 2,800 × g for 30 min at 4 °C. The supernatant was placed into a clean tube, filtered (Millipore® 0.45 μm) and placed in a sealed vial for HPLC analysis. The injection volume used was 20 μL for all brain samples. The quantity of vanillic acid was calculated by comparing the peak area ratio from tissue samples of treated animals with those of the corresponding concentration standards of vanillic acid in acetonitrile injected directly into the HPLC system.

### Tissue collection and sample preparation

For tissue analysis (n ± 8 mice/group), mice were perfused transcardially with 4% ice-cold paraformaldehyde, and the brains were postfixed for 72 h in 4% paraformaldehyde and transferred to 20% sucrose for 72 hr. The brains were frozen in O.C.T. compound (A.O., USA), and 14-μm coronal sections were cut using a CM 3050 C cryostat (Leica, Germany). The sections were thaw-mounted on probe-on plus charged slides (Fisher, Rockford, IL, USA).

### Immunofluorescence staining

Immunofluorescence staining was performed according to previously reported methods with minor modifications^77^. Briefly, slides containing either HT22 cells or the brain tissues sections (cortex and hippocampus regions of mice) were washed twice for 10 min each in 0.01 M PBS and incubated for 1 h in blocking solution containing 2% normal bovine serum (Santa Cruz Biotechnology), according to the antibody treatment, and 0.3% Triton X-100 in PBS. After blocking, the slides were incubated overnight at 4 °C with anti-p-NF-KBp65, anti-p-Akt, anti-8-Oxo-dG, anti-Aβ, anti-PSD95, anti-Iba-1 and anti-GFAP antibodies diluted 1:100 in blocking solution. Following this step, the sections were incubated for 2 h with fluorescein isothiocyanate (FITC)-labeled (green) or TRITC labeled (red) secondary antibodies (1:50). The slides were then counterstained with 40,6-diamidino-2-phenylindole (DAPI) for 10 min and mounted with the Prolong Anti-Fade Reagent (Molecular Probes, Eugene, OR, USA). Staining patterns were examined using a confocal laser-scanning microscope (FlouviewFV 1000) and were evaluated by an examiner blind to the treatment groups.

### Fluoro-Jade B staining

Fluoro-Jade B staining was performed according to the manufacturer’s protocol (Millipore, USA, Cat. #AG310, Lot #2159662) and as previously reported^79^. After air drying the specimens overnight, the slides were immersed for 5 min in a solution containing 1% sodium hydroxide and 80% ethanol. Following this, the slides were immersed in 70% alcohol and distilled water for 2 min each. The specimens were then transferred into a solution of 0.06% potassium permanganate for 10 min, rinsed with distilled water and immersed in a solution of 0.1% acetic acid and 0.01% Fluoro-Jade B for 20 min. These slides were then washed with distilled water and were allowed to dry for 10 min. The glass cover slips were mounted using DPX nonfluorescent mounting medium, and the images were acquired using a confocal laser scanning microscope (FV 1000, Olympus, Japan).

### Statistical analysis

The western blots were scanned and analyzed via densitometry using the Sigma Gel System (SPSS Inc.). The density values are expressed as the mean ± standard error of the mean (S.E.M.). The Image-J software was used for quantitative immunohistological analysis. A one-way analysis of variance (ANOVA) followed by a two-tailed independent Student’s t-test and Tukey’s multiple comparison test were used for comparisons among the treatment and control groups. The calculations and graphs were generated with Prism 5 software (GraphPad Software, In., San Diego, CA, USA). P values < 0.05 were considered to be statistically significant: ^#^significantly different from the vehicle treated control group, *significantly different from Aβ_1-42_-treated groups. *P < 0.05, and **P < 0.01; ^#^P < 0.05, and ^##^P < 0.01.

## Additional Information

**How to cite this article**: Amin, F. U. *et al*. Vanillic acid attenuates Aβ_1-42_-induced oxidative stress and cognitive impairment in mice. *Sci. Rep.* **7**, 40753; doi: 10.1038/srep40753 (2017).

**Publisher's note:** Springer Nature remains neutral with regard to jurisdictional claims in published maps and institutional affiliations.

## Supplementary Material

Supplementary Information
