# Supplementary material for: Vanillic acid attenuates Aβ1-42-induced oxidative stress and cognitive impairment in mice
Source: Sci Rep. 2017 Jan 18;7:40753. doi: 10.1038/srep40753 (PMC5241654; doi:10.1038/srep40753)

Vanillic acid attenuates $A\beta_{1-42}$-induced oxidative stress and cognitive impairment in mice

Faiz Ul Amin, Shahid Ali Shah, Myeong Ok Kim*

Department of Biology and Applied Life Science (BK 21), College of Natural Sciences,

Gyeongsang National University, Jinju, 660-701, Republic of Korea

* Corresponding author

Myeong Ok Kim, Prof. Ph.D.

Head of Neuroscience Pioneer Research Center,

Department of Biology and Applied of Life Science, College of Natural Sciences,

Gyeongsang National University, Jinju, 660-701, South Korea

Tel.: +82-55-772-1345 Fax: +82-55-772-1349

E-mail:mokim@gnu.ac.kr

Supplementary Figure

Supplementary Figure 1

(A)

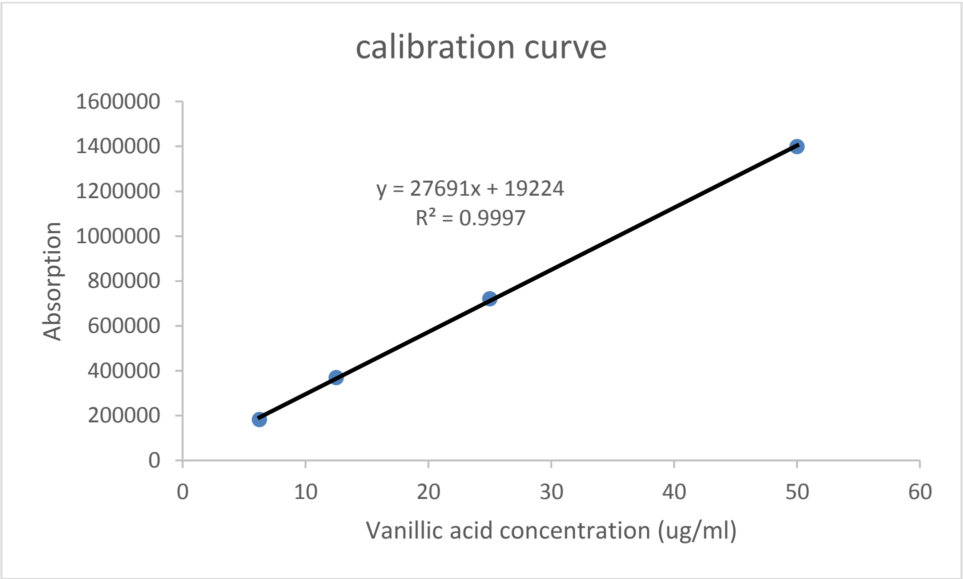

(B)

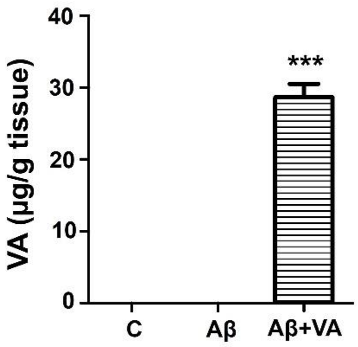

Supplementary Figure 1. (A) Calibration curve for vanillic acid. **(B)** Vanillic acid distribution in the brain after one week of daily administration of VA 30mg/kg, i.p. The values are depicted ± SD of five animals per group. ***$P < 0.001$.

Supplementary informations

Full Length Gels and their Legend

Figure 2

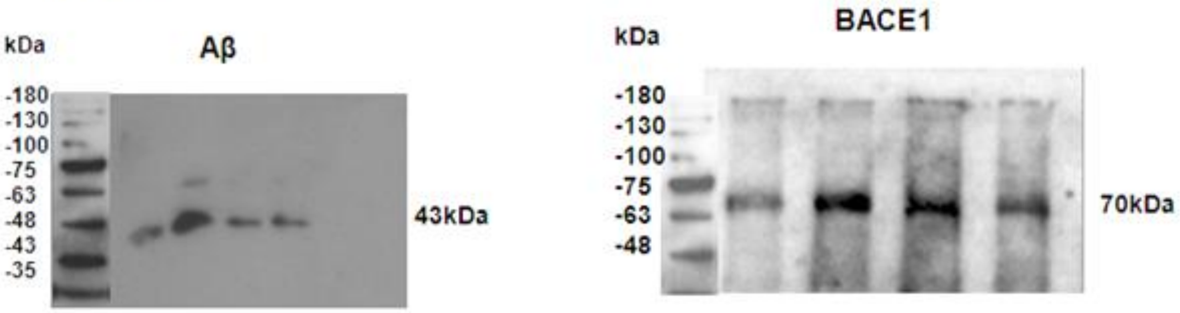

Figure Legend 2. Vanilic Acid alleviated Aβ accumulation and β-site APP cleaving enzyme 1 (BACE-1) overexpression in mice brain homogenates. **(A)** The whole gels immunoblots of Aβ (43kDa) and BACE- 1 (70kDa) protein expressions in the mice brain following Aβ and VA administration. The bands were cropped at the exact kDa and are shown in the main manuscript and quantified using Sigma Gel software, and the differences are represented by a histogram.

Figure 3

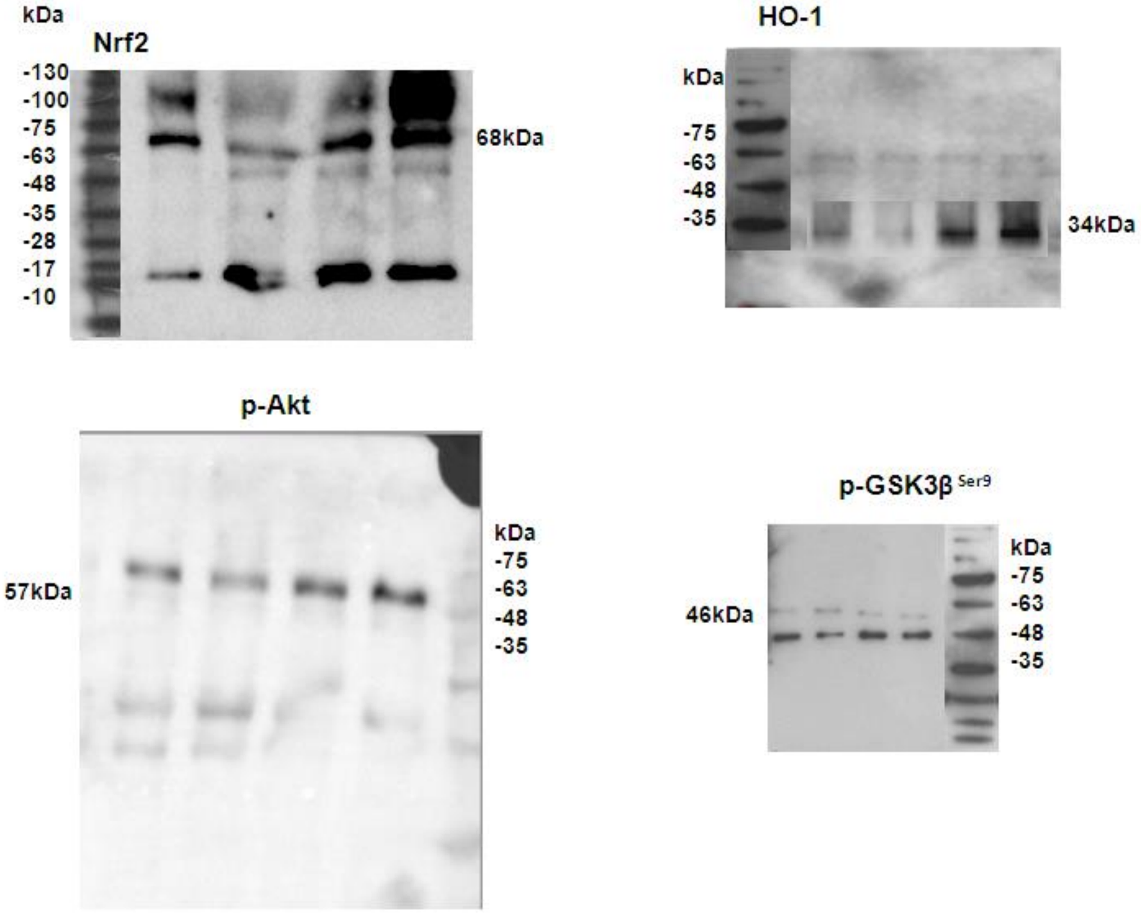

Figure Legend 3. (D) Vanillic acid treatment stimulates Akt/GSK3β/Nrf2/HO-1 pathway in the brain of Aβ_{1-42}-treated mice. The whole gels immunoblots of Nrf2 (68kDa), HO-1(34kDa), p-Akt (57kDa), GSK3β (46kDa) protein expressions in the mice brain following Aβ and VA administration. The bands were cropped at the exact kDa and are shown in the main manuscript and quantified using Sigma Gel software, and the differences are represented by a histogram.

Figure 4

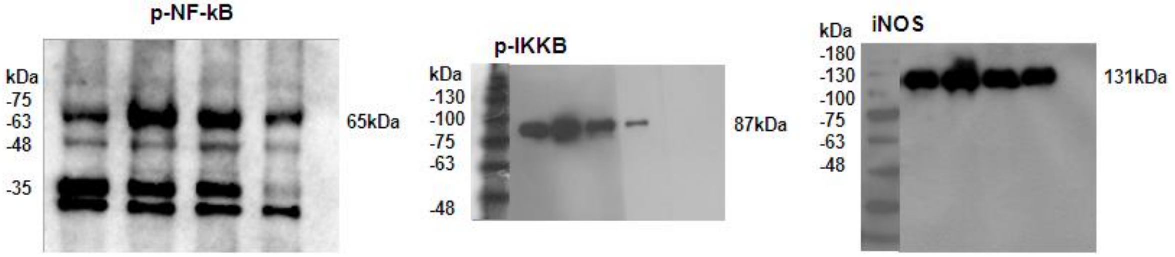

Figure Legend 4. (C) The whole gels immunoblots of p-NF-κB (65kDa), p-IKKβ (87kDa) and iNOS (131kDa) protein expressions in the brain of A$\beta_{1\text{-}42}$-treated mice. The bands were cropped at the exact kDa and are shown in the main manuscript and quantified using Sigma Gel software, and the differences are represented by a histogram.

Figure 5

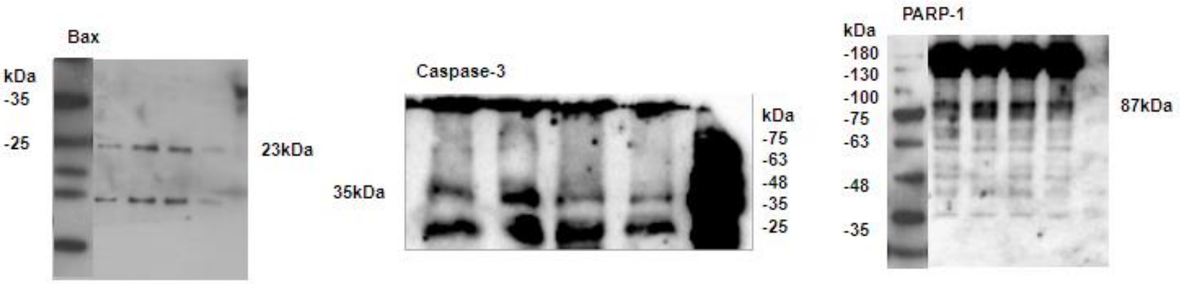

Figure Legend 5. (A) The whole gels immunoblots of Bax (23kDa), caspase-3 (35kDa) and cleaved PARP-1(87kDa) protein expressions in the brain of $A\beta_{1-42}$-treated mice. The bands were cropped at the exact kDa and are shown in the main manuscript and quantified using Sigma Gel software, and the differences are represented by a histogram.

Figure 6

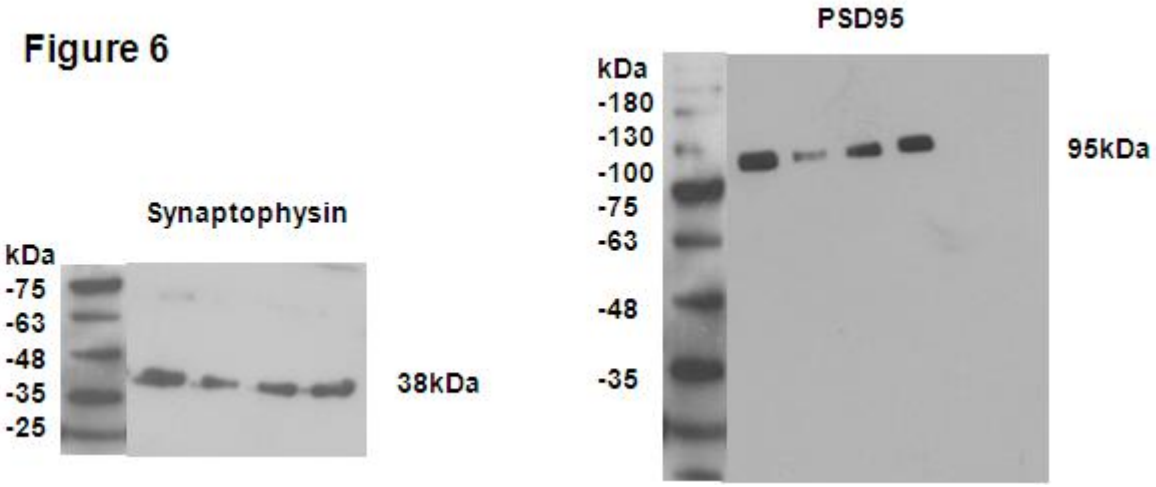

Figure Legend 6. (A) The whole gels immunoblots of Synaptophysin (38kDa) and PSD95 (95kDa) protein expressions in the brain of A$\beta_{1\text{-}42}$-treated mice. The bands were cropped at the exact kDa and are shown in the main manuscript and quantified using Sigma Gel software, and the differences are represented by a histogram.

Multiple Exposure Gels (Immunoblots)

Figure 2

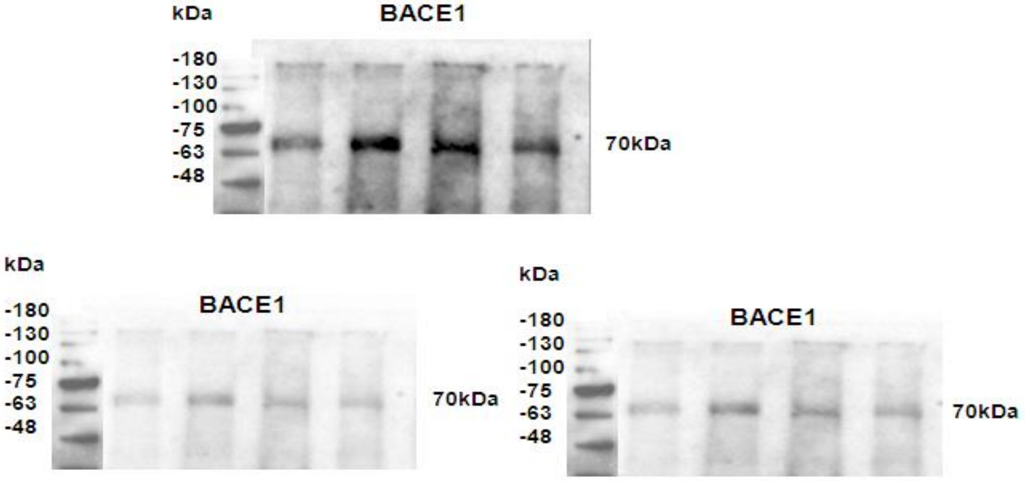

Figure 3

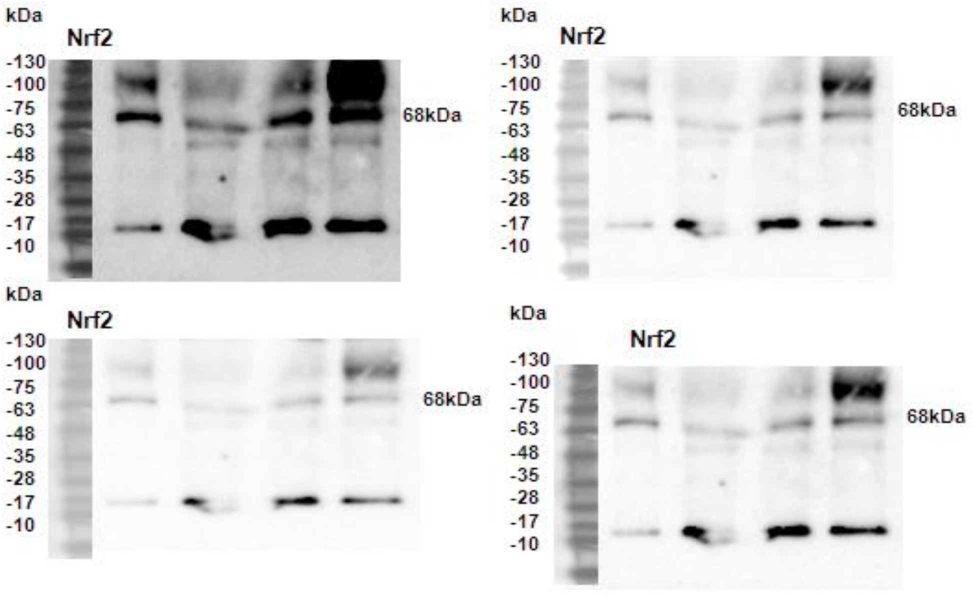

Figure 3

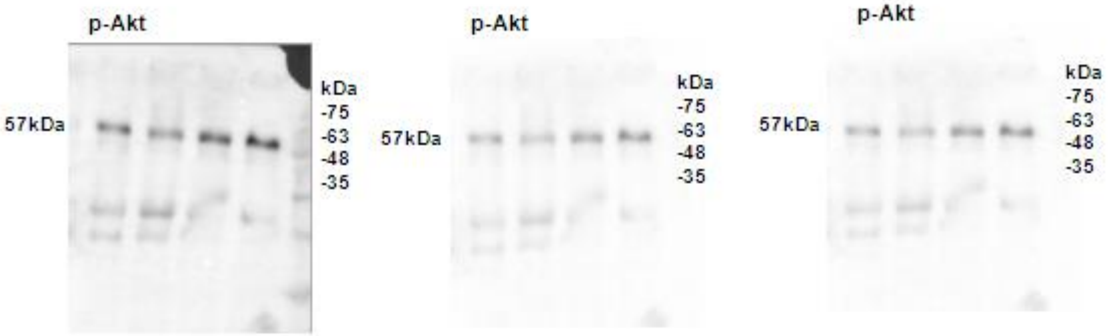

Figure 4

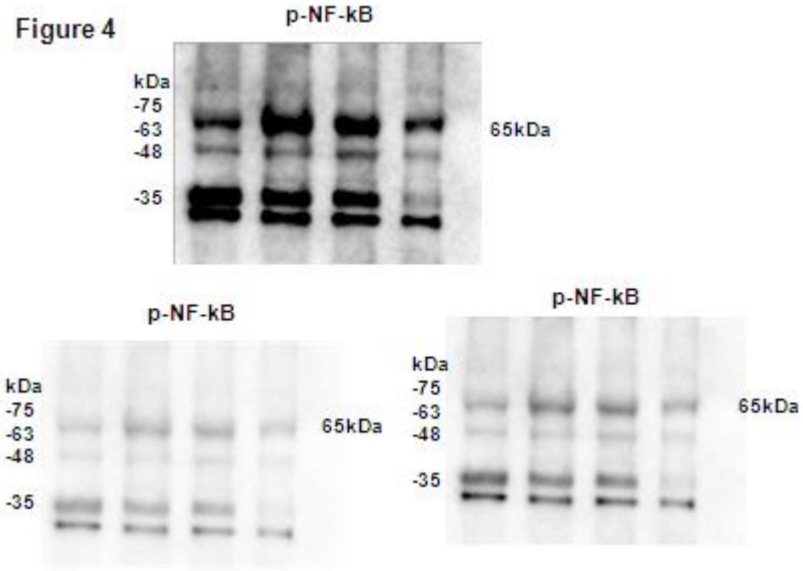

Figure 5

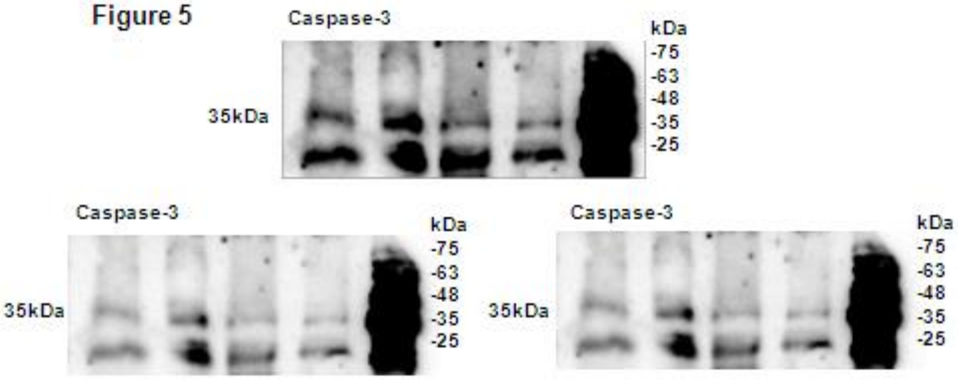

Figure 5

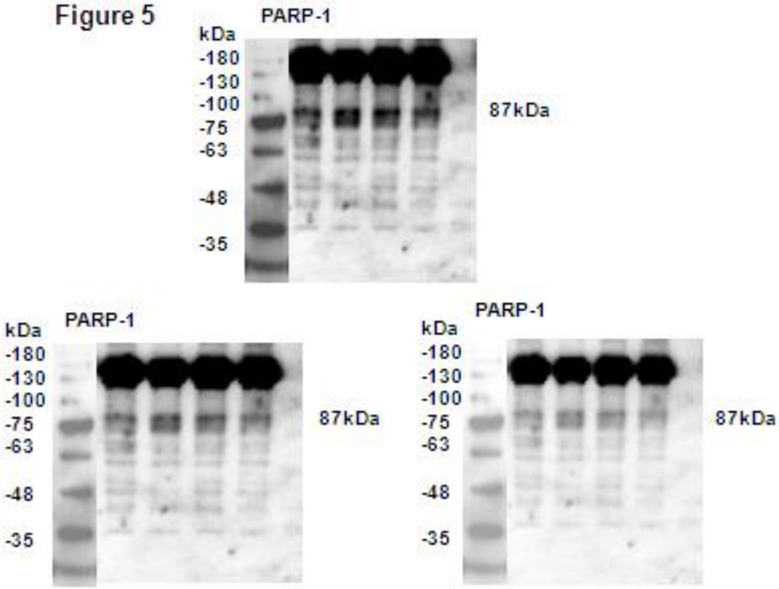

Supplement: Supplementary Information [file srep40753-s1.pdf]
